# Navigating Stress: Exploring the Role of Empathy in Caregiving

**DOI:** 10.1002/dev.70177

**Published:** 2026-06-16

**Authors:** Hannah Spencer, Peter A. Bos, Sabine van der Asdonk, Lenneke R. A. Alink, Renate S. M. Buisman

**Affiliations:** ^1^ Institute of Education and Child Studies Leiden University Leiden The Netherlands

**Keywords:** acute stress, caregiver sensitivity, empathy, infant simulator, Trier Social Stress Test

## Abstract

Stress can negatively impact caregiving behavior; while chronic stress is consistently associated with reduced caregiver sensitivity, evidence regarding acute stress remains mixed. This preregistered study examined the causal effects of acute psychosocial stress on caregiver sensitivity with an experimental pseudorandomized design. Female participants (*N* = 130, *M*_age_ = 21.16, SD = 1.65) were assigned to either an adapted Trier Social Stress Test or a control condition, and then interacted with an infant simulator while their caregiver sensitivity was observed. We also investigated the role of situational empathic behavior and trait empathic concern in the relationship between acute stress and caregiver sensitivity. Structural equation modeling demonstrated that acute stress did not significantly decrease caregiver sensitivity. Furthermore, situational empathic behavior did not explain how acute stress affected caregiver sensitivity, nor did trait empathic concern influence how acute stress affected situational empathic behavior. However, trait empathic concern was positively associated with caregiver sensitivity, affirming that empathy is fundamental to caregiving. We discuss that effects of acute stress may be more subtle compared to chronic stress and highlight the importance of sensitive measurement instruments in continued research, which will help us to further understand how stress impacts caregiving and, consequently, child outcomes.

## 1 Introduction

Caregiver sensitivity—characterized by the ability to perceive, interpret, and respond to children's signals and needs promptly and adequately—plays a fundamental role in promoting secure parent–child attachment and fostering children's social‐emotional and cognitive development (Ainsworth et al. 1978; Bakermans‐Kranenburg et al. 2003; Bowlby 1988; Cooke et al. 2022; Madigan et al. 2024). Stress can increase the risk that caregivers adopt less sensitive parenting practices, which in turn may negatively affect children's development (Belsky 1984; Belsky and Jaffee 2015; Masarik and Conger 2017; McEwen 2017). While the detrimental effects of chronic and cumulative stress are well documented, the impact of acute stress remains less clear. Studies indicate that acute stress may diminish caregiver sensitivity, leave it unaffected, or possibly even promote it (Beckerman et al. 2020; Bruinhof et al. 2025; Doan et al. 2022; Leerkes et al. 2015, Leerkes et al. 2016; Park and Johnston 2020; Probst et al. 2017; Tronick et al. 2021). These mixed findings raise important questions about how and why acute stress may influence caregiving behavior, and which underlying mechanisms are involved. Understanding these processes is crucial for informing interventions aimed at supporting caregiver sensitivity. To address this gap, the current study employed an experimental pseudorandomized design to examine the causal effects of acute psychosocial stress on caregiver sensitivity.

In particular, we focused on empathy as a potential pathway linking stress to caregiver sensitivity. Empathy is composed of affective and cognitive components that allow individuals to share and understand another person's affective state (De Waal and Preston 2017; Decety and Jackson 2004). Empathy is therefore crucial to understand a child's signals and cues, which is necessary to respond to them appropriately. Indeed, parenting interventions that include components targeting empathic behavior improve overall parenting quality and promote positive child outcomes (Havighurst et al. 2010; Juffer et al. 2017). Furthermore, parents who accurately interpret emotions and their children's mental state engage in more sensitive care (Havighurst et al. 2010; Zeegers et al. 2017), while reduced empathic abilities are associated with negative child attributions and a risk for maladaptive parenting practices (Buisman et al. 2024; Rodriguez 2013; Rodriguez et al. 2019, 2020). Since situational empathic behavioral responses are also susceptible to stress, they may help us explain how acute stress influences caregiving. Furthermore, initial evidence suggests that individual differences in trait empathic concern may influence how stress affects individuals, which could help explain mixed findings in the literature (Nitschke and Bartz 2023).

### 1.1 Stress and Its Effect on Caregiving and Empathic Behavior

Research has consistently shown that chronic stress and the accumulation of stressors, including low socioeconomic status, poor relationship quality, single parenthood, and a history of childhood maltreatment, can significantly undermine parenting practices and diminish caregiver sensitivity (Booth et al. 2018; Buisman et al. 2018, Buisman et al. 2019; Crnic and Low 2002; Savage et al. 2019). Social Information Processing (SIP) models constitute a useful framework to understand how stress can affect caregiving and empathic behavior. According to SIP theory, individuals process and respond to social information through a series of steps, including the perception and interpretation of these signals and cues, followed by the selection and execution of behavioral responses (Crittenden 1993; Milner 2003). Pre‐existing schemata, including expectations of one's children and oneself, affect how social information is processed and thus influence how individuals respond in caregiving contexts (Milner 2003). In response to a stressor—a physical, psychological, or environmental factor that is perceived as challenging or potentially threatening—cognitive load increases, which can lead to errors or biases in how individuals process, interpret, and respond to social information (Azar et al. 2008; Crittenden 1993; Milner 2003). In caregiving contexts, this may lead to decreased awareness of children's cues or misinterpreting their emotions or behavior, such as incorrectly attributing defiance to a child's innocent behavior (Azar et al. 2008; Beckerman et al. 2020; Milner 2003). Such stress‐related disruptions in the processing of social information increase the risk for maladaptive caregiving practices and, in severe cases, child maltreatment (Milner 2003).

Neuroscientific research further elucidates how stress can impair caregiving practices and empathic behavior. In response to stressors, the body's stress response initiates a cascade of physiological changes that prepare an individual to cope with the stressor. These changes include an increase in heart rate, respiration, and the secretion of (nor)adrenalin and cortisol, which provides a boost in energy by increasing glucose availability (Chrousos 2009; O'Connor et al. 2021). These changes also affect cognitive processes, influencing how individuals appraise and attend to their surroundings. When an individual experiences stress, attentional processes become more selective and vigilant, prioritizing salient and potentially threatening stimuli, while reducing distractions from irrelevant sources (de Kloet and Joëls 2023; Hermans et al. 2014; Schwabe et al. 2022). Furthermore, memory retrieval, working memory, and cognitive flexibility become impaired, leading to a shift from cognitively demanding to more habitual and self‐centered stress‐coping mechanisms that are energy efficient. While this facilitates quicker responses to stressors—also referred to as the “fight‐or‐flight” response—it can also reduce rational decision‐making, increase aggressiveness, and make it challenging to understand other's emotions or intentions (de Kloet and Joëls 2023; Faber and Häusser 2022; Hermans et al. 2014; Nitschke et al. 2022; Nitschke and Bartz 2023; Sandi and Haller 2015; Schwabe et al. 2022). These disruptions under stress can therefore undermine empathic behavior and the ability to respond sensitively to children's signals and needs (Azar et al. 2008; Belsky and Jaffee 2015; McEwen 2017; Milner 2003).

However, the effect of acute stress, which is temporary and situational, is less clear. Some studies suggest that acute stress also negatively impacts caregiving. For instance, increased stress responses during challenging parenting situations, such as interacting with a crying infant, can shift focus from child‐oriented to self‐oriented processes and compromise sensitive caregiving behavior (Leerkes et al. 2015, Leerkes et al. 2016). An experimental study also demonstrated that acute stress can lead to less adaptive parenting practices during parent–child feeding interactions (Doan et al. 2022). Similarly, experimental studies demonstrated that acute stress can undermine key situational empathic behaviors, or state measures of empathy, including emotion contagion and empathic responses to other's pain (Nitschke et al. 2020; Nitschke and Bartz 2023; Tang et al. 2024). Importantly, though, findings on the effects of acute stress on caregiving and empathic behavior are mixed. In fact, two randomized controlled trials induced acute stress in mothers prior to mother‐infant interactions and found no effects on maternal behavior, including maternal sensitivity (Bruinhof et al. 2025; Tronick et al. 2021). Furthermore, experimentally induced acute stress did not affect harsh parenting practices measured in response to vignettes or negative parenting attributions (Beckerman et al. 2020; Park and Johnston 2020). With regard to empathy, studies demonstrate similar negligible effects of acute stress on empathic behaviors, including emotion recognition (Nitschke and Bartz 2023; Smeets et al. 2009; Wingenfeld et al. 2018). Interestingly, other studies showed that acute stress is related to increased motivation to care for infants and enhanced abilities to identify emotions and act more prosocially, particularly toward socially close others (Nitschke et al. 2022; Nitschke and Bartz 2023; Probst et al. 2017). These mixed findings suggest that the effects of acute stress are complex and may vary across individuals and contexts. For some individuals, acute stress may not have negative effects on empathic behavior at all. For others, it might facilitate prosocial behavior by promoting empathic behavior and affiliation with others (Faber and Häusser 2022; Tomova et al. 2017). This process may be explained by the “tend‐and‐befriend” theory, which posits that humans, and women in particular, use social relationships to cope with stress, adaptively tending to offspring and affiliating with others for protection and stress alleviation (Taylor 2012; Taylor et al. 2000). Under this model, stress may actually enhance empathic behavior and caregiving in certain contexts.

### 1.2 Trait Empathic Concern As a Moderator

Trait empathic concern, or one's general tendency to experience “other‐oriented” feelings of compassion and concern (Davis 1983), may explain differences in how individuals respond to acute stress (Nitschke and Bartz 2023). As stated previously, the acute stress response induces cognitive changes, leading to a shift from cognitively demanding to more habitual, energy‐efficient stress coping mechanisms (de Kloet and Joëls 2023; Hermans et al. 2014; Schwabe et al. 2022). Individuals with higher levels of trait empathic concern may be more likely to engage in, and possibly even show enhanced, situational empathic behavior such as emotion contagion and recognition during stress. In contrast, those with lower levels of trait empathic concern may be more susceptible to stress‐related impairments in these behaviors. Initial evidence suggests that individuals with higher levels of trait empathic concern exhibit increased contagion of stress responses when observing others undergoing acute stress (Buchanan et al. 2012; Engert et al. 2014; Nitschke and Bartz 2023). Additionally, trait empathic concern has been shown to moderate the effects of acute stress on prosocial behavior. During an economic game, acute stress responses led to increased generosity in individuals with high levels of trait empathic concern, while those with low levels of trait empathic concern showed more self‐centered behavior under stress (Azulay et al. 2022). Therefore, individuals with lower levels of trait empathic concern may be more inclined to show diminished situational empathic behavior in response to acute stress, while those with higher levels of trait empathic concern may be more prone to enhanced situational empathic behavior. Such differential effects of acute stress on situational empathic behavior may help account for the mixed findings in the literature on how acute stress influences caregiving practices.

### 1.3 The Current Study

**FIGURE 1:**
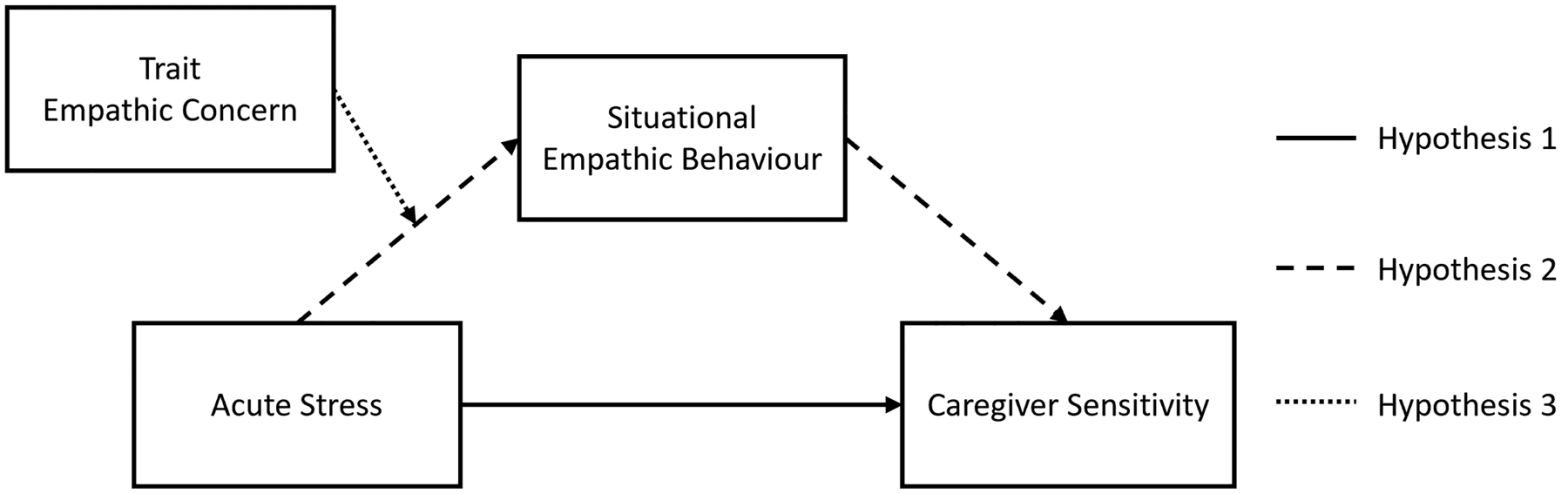
A visualization of our hypotheses.

The current study aimed to contribute to a more comprehensive understanding of how acute stress impacts caregiver sensitivity by examining how empathy may elucidate this relationship. Specifically, we examined the causal effects of acute psychosocial stress on caregiver sensitivity. Participants were assigned to either an adapted version of the Trier Social Stress Test (TSST) or a control condition (Kirschbaum et al. 1993). We (1) hypothesized that participants assigned to the TSST would show lower subsequent levels of caregiver sensitivity compared to those in the control condition. Additionally, we (2) examined situational empathic behavior with two standardized tasks and hypothesized that situational empathic behavior would (partially) explain how acute stress affects caregiver sensitivity. Considering the heterogeneity in research findings on the effects of acute stress, we also accounted for participant's levels of trait empathic concern. We (3) hypothesized that individuals with lower levels of trait empathic concern would be more likely to exhibit diminished situational empathic behavior in response to stress, while participants with higher levels of trait empathic concern were expected to show increased situational empathic behavior (see Figure 1 for a visualization of our hypotheses).

## 2 Materials and Methods

### 2.1 Participants

A total of 130 female participants were included in the study (*M*_age_ = 21.16, SD = 1.65, range = 18.35–25.48 years). Only nulliparous females were included to ensure a homogeneous sample, since sex differences are well known to influence responses to acute stressors, and parenthood may influence responses to an infant simulator (Liu et al. 2017; Plieger and Reuter 2020; Sinisalo et al. 2022). Prior to study participation, all participants were screened for the following inclusion criteria: (1) aged between 18 and 25 years; (2) enrolled as a student in secondary vocational education, higher professional education, or university education in a non‐pedagogical degree (to avoid familiarity with the infant simulator); (3) does not have children and no current or past pregnancy; and (4) no psychiatric disorder or physical impairment that may limit data collection. The sample size was based on a priori power calculations conducted in G*power 3.1.9. To test the mediation effect that empathic behavior (partially) explains the effect of acute stress on caregiver sensitivity, we determined that a sample size of 100 participants was necessary based on an *F*‐test power calculation for a multiple regression with two predictors (stress condition assignment and situational empathic behavior), an *α* of 0.05, an expected small effect size (*f* ^2^ = 0.10), and a power of 0.80. To test the moderation effect of trait empathic concern on the association between acute stress and situational empathic behavior, we determined that a sample size of 114 participants was necessary based on an *F*‐test power calculation for a multiple regression with three predictors (stress condition assignment, trait empathy, and their interaction), an *α* of 0.05, an expected small effect size (*f* ^2^ = 0.10), and a power of 0.80. Accounting for a possible dropout rate of 10%, we aimed to include a total of 126 participants (63 per condition). In total, 63 participants were assigned to the stress condition, and 67 participants were assigned to the control condition. Due to technical errors during data collection, we included four additional participants, who were already signed up to participate, to minimize the risk of substantial missing data. Participants in both conditions did not differ in age, body mass index (BMI), education level, or use of hormonal contraceptives (see Table 1).

**TABLE 1:** Overview of demographic data for participants in the stress and control condition.

	Stress condition (*n* = 63) *M* (SD) or *n* (%)	Control condition (*n* = 67) *M* (SD) or *n* (%)	Between‐group test statistics
Age in years	21.23 (1.70)	21.10 (1.61)	*t* (126.24) = −0.42, *p* = 0.67
BMI	23.32 (3.83)	22.65 (3.67)	*t* (126.59) = −1.02, *p* = 0.31
Education level			*Χ* ^2^(2) = 0.27, *p* = 0.87
University education	51 (80.95)	52 (77.61)	
Higher professional education	10 (15.87)	12 (17.91)	
Secondary vocational education	2 (3.17)	3 (4.48)	
Hormonal contraceptive use			*Χ* ^2^(2) = 0.60, *p* = 0.74
No hormonal contraceptives	30 (47.62)	29 (43.28)	
Hormonal contraceptive use (oral contraceptives/hormonal IUD)	25 (39.68)	31 (46.27)	
Unknown (prefer not to say/other)	8 (12.70)	7 (10.45)	

Abbreviations: BMI = body mass index; IUD = intrauterine device

### 2.2 Procedure

The current study was preregistered in the OSF registries on October 08th, 2024, after data collection was completed and prior to performing statistical analyses, doi: 10.17605/OSF.IO/RU98J. The study was conducted in accordance with the latest version of the Declaration of Helsinki and approved by the Institute of Education and Child Studies Ethics Committee of Leiden University (ECPW2021‐296). Data were collected during a lab visit at the Faculty of Social and Behavioural Sciences at Leiden University, which lasted approximately 1.5 h. Lab visits started with a general explanation, after which participants provided written informed consent. They then completed a set of questionnaires measuring parental care motivation, empathy, and daily hassles, and an Empatica E4 wristband was fitted for physiological data acquisition as part of a larger project. This was followed by a baseline period, which consisted of a 30‐s discussion of a series of neutral pictures and a 5‐min relaxation video. Immediately after the baseline period, participants provided the first saliva sample. Electrodes were then fitted to measure automatic facial muscle responses during computer tasks. Following this, participants underwent either a stress manipulation (TSST) or a control condition. Both conditions were interrupted to complete two computer tasks measuring situational empathic behavior and to take care of an infant simulator to assess caregiver sensitivity. Finally, two further saliva samples were provided. See below for detailed descriptions of procedures implemented in the current study.

We used a pseudorandomized method to alternately assign participants to either the stress or control condition. The assignment followed the order of trial entrants, after participants were screened for inclusion criteria. The screeners were blind to the assignments of previous participants to ensure they could not influence the allocation of new participants. Participants remained unaware of the specific manipulation (stress or control) they were assigned to until the debrief at the end of the lab session. Due to the study's nature, research members involved in data collection in the lab could not be blinded. Research members who were involved in data processing and coders of caregiver sensitivity were blinded to condition assignment. To test if the stress manipulation was successful, we conducted a manipulation check by comparing perceived stress responses between the stress and control conditions. Additionally, we compared physiological stress responses between the stress and control conditions with changes in cortisol concentrations in saliva over time in response to the manipulation (Pruessner et al. 2003).

### 2.3 Stress Manipulation and Measurement Instruments

#### 2.3.1 Acute Stress Condition

In the acute stress condition, participants underwent an adapted version of the Trier Social Stress Test (TSST; Kirschbaum et al. 1993). At the start of the TSST, a research member, portrayed as a “committee member,” entered the room wearing a lab coat while behaving coldly and distantly. They instructed the participant to prepare a 5‐min speech as if they were applying for a job. The speech would be delivered to a committee acting as a job interview panel, who would evaluate style, content, and behavior while observing from behind a one‐way mirror. Each participant received a job description that matched their educational level.

After 5 min of preparation, participants began their speech. One minute in, they were interrupted and asked to complete two computer tasks measuring situational empathic behaviors (for details, see below). Upon completing these tasks, participants resumed their speech for an additional 5 min. Midway through the speech, the committee member returned and posed a question: *The committee would like to learn more about your weaknesses*. Following the speech, participants were asked to verbally solve a difficult math problem. After 1 min, they were interrupted again; this time to care for an infant simulator for 10 min to measure caregiver sensitivity (for details, see below). Before participants were introduced to the infant simulator, they were informed that they would continue with the math problem afterward and were also asked to prepare a job‐related case study to present to the committee. However, after caring for the infant simulator, participants were informed that their study participation had concluded, and they received a debrief.

#### 2.3.2 Control Condition

The control condition consisted of an adapted version of the placebo TSST (e.g., Het et al. 2009). First, participants were asked to complete a questionnaire for 5 min about holidays and hobbies. Afterward, a warm and friendly research member held an informal conversation with participants about their last holidays. One minute into this conversation, participants were asked to complete the two computer tasks measuring situational empathic behaviors, after which the conversation was resumed. After the 5‐min conversation, participants were asked to play a game on a tablet. After 1 min, participants were asked to care for the infant stimulator. Finally, participants were informed that their study participation had concluded, and they received a debrief.

#### 2.3.3 Perceived Stress Responses

We evaluated perceived stress responses using visual analogue scales (VAS). Participants were asked to rate their stress levels on a VAS ranging from 0 to 100. This question was administered prior to the start of the stress or control condition (T1) and prior to conducting the computer empathy tasks (T2). Perceived stress responses were calculated using residualized change *Z*‐scores, with the perceived stress VAS score at T1 as the predictor variable and the perceived stress VAS score at T2 as the outcome variable. Perceived stress responses were missing for two participants (one participant in the stress condition, and one in the control condition).

#### 2.3.4 Cortisol Responses

We examined time‐dependent changes in cortisol concentrations using three saliva samples collected via passive drooling in 2‐mL collection tubes (Sarstedt AG & Co. KG, Nümbrecht, DE). The first sample was collected immediately after the baseline period and prior to the start of the stress or control condition. The second sample was collected after the participant cared for the infant simulator, which was on average 63 min after the first sample was collected (SD = 7 min). The third sample was collected after the debriefing at the end of study participation, which was on average 10 min after the second sample was collected (SD = 2 min). Saliva samples were stored at −80°C until cortisol concentrations were quantified by Dresden LabService GmbH using liquid chromatography with coupled tandem mass spectrometry (LC‐MS/MS). The intra‐assay and inter‐assay coefficients of variation (CV) were 3.3% and 8.8%, respectively. To assess cortisol changes over time, we calculated the “Area under the curve with respect to increase” (AUC_i_) using the formula defined by Pruessner et al. (2003). AUC_i_ values could not be calculated for 16 participants (seven participants in the stress condition, and nine in the control condition, no difference between conditions, *Χ* ^2^(1) = 0.02, *p* = 0.89) because of missing cortisol concentrations at differing sampling times due to empty or missing collection tubes.

#### 2.3.5 Situational Empathic Behavior

##### 2.3.5.1 Affective Images Task

Participants completed two computer tasks to measure situational empathic behavior. During the first task, participants were presented with 30 affective black and white images of children in positive, negative, and neutral contexts. Positive images showed children in positive social interactions (like reading with a parent), negative images showed children in socially distressing scenes (such as a begging homeless child), and neutral images showed children in neutral situations (like playing on a computer with a neutral facial expression). Images were randomly presented for 2000 ms and were preceded by a 1000‐ms fixation cross. After image presentation, participants rated their compassion and positive affect in response to the image on a scale from 1 (*not at all*) to 9 (*a lot*). Prior studies conducted by our research group have validated this task as an effective measure of empathic behavior (Spencer et al. 2022). During the task, electromyography (EMG) activity was recorded to measure automatic facial muscle responses by placing bipolar electrodes over the left zygomaticus major (ZYG) to measure smiling responses, and over the left corrugator supercilii (COR) to measure frowning responses (Fridlund and Cacioppo 1986). The ground electrodes consisted of the active common mode sense (CMS) and passive driven right leg (DRL) electrodes that were placed over the midline of the forehead. Electrodes were placed directly after participants provided the first saliva sample and prior to the start of the stress or control condition. EMG activity was recorded at a sampling rate of 2048 Hz using the Biosemi Active Two system.

EMG data reduction was conducted with Brain Vision Analyzer 2.2. Raw EMG data underwent band‐pass filtering between 30 and 500 Hz with a 24 dB roll‐off per octave. For each image presentation, the data were segmented into epochs spanning from −1000 to 2000 ms, aligned with image onset. Next, the data were rectified, averaged into 250‐ms intervals, and exported into R Version 4.4.1 (R Core Team 2024) for further processing. Normalization was performed by calculating the proportion of mean rectified activity within each 250‐ms interval relative to the mean rectified baseline activity (−1000 to 0 ms) for each stimulus presentation. This normalization allowed us to express EMG activity at each timepoint relative to the average baseline, with a value of 1.00 indicating equal activity. Trials were rejected as artifacts if the mean rectified baseline EMG activity or mean normalized poststimulus onset (0–2000 ms) EMG activity deviated by ±3 standard deviations from the mean activity within subjects. Extreme EMG activity in remaining trials, defined as ±3 standard deviations from the mean activity across subjects, was treated as extreme outliers and excluded from further analysis. In total, 4.50% of COR activity and 6.60% of ZYG activity were omitted from analyses due to detection as artifacts or extreme outliers. Due to technical errors, EMG data were not collected correctly, and were therefore missing, for 24 participants (12 participants in the stress condition, and 12 in the control condition).

We computed situational empathic behavior by extracting effect estimates per participant from linear mixed‐effects analyses using the lmerTest (Kuznetsova et al. 2014) and lme4 (Bates et al. 2015) packages in R Version 4.4.1 with maximum‐likelihood estimation and bound optimization by quadratic approximation with a set maximum of 100,000 iterations. For each of our outcome measures (compassion and positive affect ratings, as well as mean ZYG and COR activity in response to positive and negative images), we included the fixed effect of condition and the random effects of participant and image, as well as the random slopes of condition over participant, into separate models. Greater effect estimates indicate a larger difference in subjective ratings and muscle activity in response to positive and negative images, reflecting enhanced empathic responses. Missing values (i.e., artifacts and outliers) were omitted from analyses, and EMG activity was log‐transformed prior to analyses due to positive skewness.

##### 2.3.5.2 Reading the Mind in the Eyes Task

The second computer task consisted of the Reading the Mind in the Eyes Task (RMET; Baron‐Cohen et al. 2001). During the RMET, participants were presented with 36 images of the eye region of different faces and were asked to choose which of four descriptions of mental states best described what the person in the image was thinking or feeling (e.g., bored, amused, or alarmed). We assessed empathic behavior by computing the number of correct items and the average response time (*RT*) to these correct items in line with previous experiences of our research group that response time might be a more sensitive measure than the number of correct responses (Buisman et al. 2024).

##### 2.3.5.3 Situational Empathic Behavior Composite Scores

We performed a Principal Component Analysis (PCA) to explore underlying factors in our measures of situational empathic behavior. In total, our two computer tasks provided us with six measures of situational empathic behavior per participant: effect estimates of (1) ZYG activity, (2) COR activity, (3) ratings of compassion, (4) ratings of positive affect in response to affective images, (5) number of correct items during the RMET, and (6) average response time to these correct items. PCA analyses revealed that a total of four components best explained our data and accounted for 84.21% of the variance. Effect estimates of compassion and positive affect ratings during the affective images task were combined into a composite score “*Empathic ratings*” by averaging their absolute values to facilitate interpretation. Higher values indicated that participants demonstrated a greater distinction between positive and negative images, reflecting enhanced empathy. Similarly, effect estimates of ZYG and COR activity were combined into a composite score “*Empathic facial reactivity*” using the same method. The number of correct items during the RMET and the average response time to these items were treated as separate components (for details, see Supporting Information).

#### 2.3.6 Caregiver Sensitivity

We assessed caregiver sensitivity by instructing participants to interact with an infant simulator, a life‐like doll resembling a young infant in appearance, size, and weight (RealCare Baby II‐Plus; Realityworks, Eau Claire, WI). Using an infant stimulator is a reliable and valid way to assess caregiver sensitivity and is also suitable for populations without children of their own (Bakermans‐Kranenburg et al. 2015; Voorthuis et al. 2013). In a lab room decorated like a living room, participants were instructed to take care of the infant simulator as if it were their own child, including careful handling such as offering neck support. They were told that the infant simulator could cry just like a real infant. They could use various objects to soothe the infant (i.e., a blanket, toys, bottle, diaper, and a second set of clothes), and could use a computer if they wanted to check their e‐mail or browse the internet. In both conditions, the simulator was programmed to be quiet for approximately 3 min, then cry for approximately 5 min, followed by being quiet for approximately 2 min. Furthermore, the infant simulator was programmed not to respond to the care provided, in line with previous research conducted by our research group (Buisman et al. 2023). Interactions with the infant simulator were videotaped and stored for later coding of caregiver sensitivity using the Ainsworth Sensitivity Scale (Ainsworth et al. 1974). The scale was slightly adapted by SvdA and LRA, both experienced coders with the Ainsworth Sensitivity Scale, to fit coding the interaction with the infant simulator (available upon request). A sensitivity score, ranging from 1 (*extremely insensitive*) to 9 (*extremely sensitive*), was assigned to the interaction. Five coders (master students in Education and Child Studies) were extensively trained by SvdA and regularly supervised to prevent coder drift. Between‐coder reliability was good: the mean intraclass correlation coefficient (ICC; single measures, absolute agreement), per pair of coders, was 0.81 (SD = 0.04, range = 0.74–0.88) on a reliability set of 20 tapes.

#### 2.3.7 Trait Empathic Concern

Trait empathic concern was assessed from the Empathic Concern Subscale of the Dutch version of the Interpersonal Reactivity Index (IRI; De Corte et al. 2007). The subscale consists of seven items that were answered on a scale from 1 (*Does not describe me well*) to 5 (*Describes me well*). An example item is as follows: “I often have tender, concerned feelings for people who are less fortunate than I am.” Internal consistency of the Empathic Concern subscale was acceptable in the current study (Cronbach's *α* = 0.76). The average score on items was used as a measure of trait empathic concern, with higher scores indicating higher levels of trait empathic concern.

### 2.4 Statistical Analyses

We tested our hypotheses by conducting linear regression analyses using the SEM function of the Lavaan package in R Version 4.4.1 (Rosseel 2012). We used the “mlr” estimator, which uses a sandwich estimator and provides robust fit indices and standard errors by implementing the T2* chi‐square statistics, which are a modification of the Satorra‐Bentler scaled chi‐square (Satorra and Bentler 1994; Yuan and Bentler 2000). We accounted for missing data by using the full information likelihood (FIML) approach. To test whether acute stress negatively affects caregiver sensitivity, we included the stress manipulation condition into our analyses as the independent variable and caregiver sensitivity as the dependent variable. The control condition was dummy‐coded as 0 and the stress condition as 1. To test if situational empathic behavior (partially) explained how acute stress affects caregiver sensitivity, we included the stress manipulation condition into our analyses as the independent variable, caregiver sensitivity as the dependent variable, and situational empathic behaviors (empathic facial reactivity, empathic ratings, number of correct answers, and the average reaction time to these correct answers during the RMET) as mediator variables in separate analyses. Finally, to test if trait empathic concern influenced how stress affected situational empathic behavior, we included the stress manipulation condition as the independent variable and situational empathic behavior as the dependent variable in separate analyses. Trait empathic concern was standardized and included in the analyses as the moderator variable.

Recognizing that the TSST does not always elicit a stress response, and some individuals even respond to the control condition, we also explored individual differences in physiological stress responses and perceived stress ratings (Azulay et al. 2022; Miller et al. 2013). Specifically, we examined how time‐dependent changes in cortisol concentrations in saliva and perceived stress ratings in response to the manipulation impacted our dependent variables. Prior to our analyses, we assessed whether relevant covariates (i.e., age, education level, BMI, and use of hormonal contraceptives) were associated with outcome measures (Montoya and Bos 2017; Pavlova and Sokolov 2022; Plieger and Reuter 2020). For each hypothesis, we corrected for multiple testing using the Benjamini–Hochberg procedure with a False Discovery Rate (FDR) of 0.10 (Benjamini and Hochberg 1995).

## 3 Results

### 3.1 Descriptive Statistics and Manipulation Check

First, we assessed whether covariates (i.e., age, education level, BMI, and use of hormonal contraceptives) were associated with outcome measures. Both age and BMI were unrelated to all outcome measures (*p*s > 0.10, see Table 2). There was a significant association between education level and cortisol response, *F* (2, 111) = 4.94, *p* = 0.01. Participants with a university education level showed a blunted decline in cortisol concentrations over time (*M* = −31.92, SD = 101.24) compared to participants with a higher professional education level (*M* = −106.22, SD = 97.98). There was no difference between participants with a secondary vocational education level and participants with a higher professional education level or university education level, *p*s ≥ 0.45. There was also a significant effect of education level on the correct number of responses on the RMET, *F* (2, 127) = 3.74, *p* = 0.02. The average number of correct answers was higher for participants with a university education level (*M* = 26.85, SD = 3.37) compared to participants with a secondary education level (*M* = 23.00, SD = 2.00). All other comparisons were not significant, *p*s ≥ 0.20. There was no significant group difference for education level on all other variables, *p*s ≥ 0.22. For use of hormonal contraceptives, there were no significant group differences on all variables, *p*s ≥ 0.07. Therefore, only the education level was taken into account as a numeric covariate with three levels when testing models with the cortisol response and the correct number of responses on the RMET.

**TABLE 2:** Overview of Pearson correlations between measures.

Measures (scaled)	1	2	3	4	5	6	7	8	9
1. Age									
2. BMI	0.12								
3. Cortisol response	0.04	−0.05							
4. Perceived stress response	0.02	0.05	0.34***						
5. Caregiver sensitivity	−0.05	−0.15	−0.10	0.14					
6. Trait empathic concern	0.11	−0.05	−0.01	0.01	0.28**				
7. Empathic facial reactivity	0.01	−0.02	−0.12	−0.05	0.16	0.16			
8. Empathic ratings	0.04	−0.04	−0.01	0.01	0.10	0.21*	0.19		
9. Correct items RMET	0.02	−0.08	0.03	−0.04	−0.05	−0.06	0.07	0.13	
10. RT correct items RMET	0.03	0.10	−0.10	0.12	0.00	−0.13	−0.07	0.00	0.21*

Abbreviations: BMI = body mass index; RMET = Reading the Mind in the Eyes Task; RT = reaction time.

*p < 0.05; **p < 0.01; ***p < 0.001.

Next, we examined correlations between continuous variables (see Table 2). Interestingly, caregiver sensitivity was significantly and positively associated with self‐reported trait empathic concern (*p* < 0.01), but not with the measures of situational empathic behavior (*p*s > 0.11). Trait empathic concern was further significantly and positively associated with empathic ratings (*p* < 0.05), but not with other measures of situational empathic behavior (*p*s > 0.09). Cortisol responses were significantly and positively associated with perceived stress ratings, indicating that participants with higher cortisol responses also reported higher perceived stress during the experiment (*p* < 0.001). Also, the number of correct items on the RMET was significantly correlated with the average RT to those correct items, suggesting that participants who took longer to respond tended to answer more items correctly. To account for this, we controlled for the number of correct responses when examining effects on RT to correct items. Finally, a manipulation check showed that our TSST stress manipulation was successful in inducing acute stress responses. Participants in the control condition showed a significantly stronger decline in cortisol concentrations over time (*t* (98.36) = −3.81, *p* < 0.001) and had lower perceived stress responses (*t* (71.81) = −11.65, *p* < 0.001) compared to participants in the stress condition.

### 3.2 Main Analyses

#### 3.2.1 Acute Stress and Caregiver Sensitivity

The regression analysis indicated no effect of stress condition on caregiver sensitivity, *B* = 0.03, *β* = 0.01, SE = 0.30, *p* = 0.91. Additional regression analyses demonstrated that individual differences in stress reactivity neither predicted caregiver sensitivity, with both cortisol responses, *B* = −0.16, *β* = −0.10, SE = 0.15, *p* = 0.28, and perceived stress ratings, *B* = 0.24, *β* = 0.15, SE = 0.13, *p* = 0.051, not significantly predicting caregiver sensitivity.

#### 3.2.2 Mediation Effect of Situational Empathic Behavior

Our mediation analyses revealed that none of our measures of situational empathic behavior significantly mediated the effect of stress condition, or individual differences in stress reactivity, on caregiver sensitivity, all *p*s ≥ 0.14. Although not statistically significant after correcting for multiple testing, results suggested a possible association between the stress condition and empathic facial reactivity during the affective images task, *B* = −0.02, *β =* −0.19, SE = 0.01, *p* = 0.04, indicating that participants in the stress condition distinguished less between positive and negative affective images compared to participants in the control condition. There were no further significant effects of stress condition, or individual differences in stress responses, on situational empathic behavior, nor were there significant effects of situational empathic behavior on caregiver sensitivity, all *p*s > 0.05.

#### 3.2.3 Moderation Effect of Trait Empathic Concern

Although not statistically significant after correcting for multiple testing, our moderation analyses suggested a possible moderation effect of trait empathic concern on the relation between perceived stress responses and average RT of correct responses during the RMET, *B* = −0.20, *β* = −0.20, SE = 0.08, *p* = 0.02. This implied that for participants with low levels of trait empathic concern, increased perceived stress responses were associated with slower responses, while for participants with high levels of trait empathic concern, perceived stress responses were not associated with response time. There was no further evidence that trait empathic concern moderated the effect between stress condition, or individual differences in stress responses, and our measures of situational empathic behavior, all *p*s > 0.07.

## 4 Discussion

In this study, we experimentally induced acute stress to investigate its causal effects on caregiver sensitivity while participants interacted with an infant simulator. Additionally, we explored how empathy might elucidate the effect of acute stress on caregiver sensitivity. Our manipulation checks confirmed that the acute stressor effectively induced stress in participants, as evidenced by blunted declines in cortisol concentrations over time and higher perceived stress responses in the stress condition compared to the control condition. Our findings did not support our initial hypotheses that: (1) acute stress impairs caregiver sensitivity, (2) situational empathic behavior (partially) explains how stress affects caregiver sensitivity, and (3) trait empathic concern influences how stress affects situational empathic behavior. However, they do offer valuable insights that warrant further investigation.

First of all, our finding that acute stress and individual differences in stress responses did not affect caregiver sensitivity contrasts with our theoretical framework, which suggested that stress impairs cognitive processes that can undermine caregiving behavior (Azar et al. 2008; Belsky and Jaffee 2015; Crittenden 1993; McEwen 2017; Milner 2003). While the detrimental effects of chronic and cumulative stress on parenting and caregiver sensitivity are well established (Booth et al. 2018; Buisman et al. 2018, Buisman et al. 2019; Crnic and Low 2002; Savage et al. 2019), the effect of acute stress on caregiving practices is less clear, with many mixed results (Beckerman et al. 2020; Bruinhof et al. 2025; Doan et al. 2022; Leerkes et al. 2015, 2016; Park and Johnston 2020; Probst et al. 2017; Reijman et al. 2016; Tronick et al. 2021). One possible explanation for these mixed results is differences in acute stress manipulations and measurements of parenting practices, making it difficult to compare findings. However, these mixed findings may also suggest that the effect of acute stress may manifest in more specific domains of caregiving—such as heightened protective or vigilant behaviors—rather than in global measures of sensitivity, especially when compared to the well‐established effects of chronic or cumulative stressors. Furthermore, acute stress may have particularly detrimental effects in high‐risk groups, such as individuals experiencing high levels of chronic or cumulative stress (Beckerman et al. 2020). To support stronger causal inferences about the role of acute, chronic, and cumulative stress (and to parse out these effects), longitudinal research designs are essential.

Importantly, our findings align with previous studies indicating that acute stress does not influence a global qualitative measure of observed caregiving behavior (Bruinhof et al. 2025; Tronick et al. 2021). This may also suggest that the effects of acute stress are too subtle to be captured by such global measures (Bruinhof et al. 2025; Tronick et al. 2021). Indeed, acute stress appears to influence more fine‐grained behaviors involved in care responses, such as the frequency and prosody of vocalizations, gaze behavior, and smiling responses (Bruinhof et al. 2025; Nitschke et al. 2020; Tronick et al. 2021; Vatheuer et al. 2021). More detailed observational micro‐coding instruments may therefore be better suited to detect subtle changes in caregiving responses following stress (Tronick et al. 2021). Moreover, participants in the current study interacted with an infant simulator programmed to cry for approximately 50% of the time and remain unresponsive to the care provided. This scenario likely posed a significant source of stress in itself, possibly limiting the observable effects of the acute stress manipulation administered beforehand. Indeed, a study recently demonstrated that stress induced by interacting with a crying infant simulator resulted in reduced parental mentalizing abilities (Malcorps et al. 2025). This source of stress may be an interesting additional avenue for future research to disentangle. For example, external sources of stress that pose a threat to a child may more likely result in protective or nurturing care responses than stress originating directly from the child being cared for (Bos et al. 2026).

Although acute stress did not directly influence caregiver sensitivity, we examined whether its potential influence might be explained through situational empathic behavior, which is key to caregiver sensitivity. While our analyses did not confirm our hypothesis, our findings do provide important considerations. First, our findings emphasize that empathic behavior cannot be condensed into a single construct. Instead, empathy comprises both bottom‐up (affective) and top‐down (cognitive and regulatory) processes (De Waal and Preston 2017; Decety and Jackson 2004). Bottom‐up processes involve automatic responses, such as emotional contagion or facial mimicry, while top‐down processes involve self‐regulation and perspective‐taking to understand others’ affective states (De Waal and Preston 2017; Decety and Jackson 2004). To include empathic behavior within our models, we had to reduce complex behavioral data into single‐effect estimates. This likely reduced the variability in our measures, particularly in automatic facial responses, which is an important index of affective empathy (Dimberg et al. 2011; Spencer et al. 2022). These facial responses are typically subtle with a rapid dynamic time‐course, and collapsing data across time and emotional valence (i.e., combining smiling and frowning responses to positive and negative affective images) may have diminished our ability to detect meaningful effects (Bos et al. 2016; Spencer et al. 2022, 2024; Tassinary and Cacioppo 1992; van Boxtel 2010). Indeed, prior research indicates that acute stress may particularly compromise smiling responses to positive affective images (Nitschke et al. 2020). Although we observed some indications that stress may compromise automatic empathic facial reactivity, with participants in the stress condition distinguishing less between positive and negative affective images compared to participants in the control condition, these effects were not significant after correcting for multiple comparisons. This may thus be due to the methodological compromises we made for statistical modeling, reducing our ability to detect meaningful effects. In addition, we found no evidence that acute stress influenced cognitive components of empathy, as measured by the RMET. This does align with previous studies reporting null effects of acute stress on emotion recognition and mental state inference (Smeets et al. 2009; Wingenfeld et al. 2018).

Finally, we found no evidence that trait empathic concern altered the effect of acute stress on situational empathic behavior. We found some indication that increased perceived stress responses may particularly disrupt social processing in participants with low levels of trait empathic concern, but not in those with high levels of trait empathic concern, as reflected in slower responses to correct RMET items. However, these effects were not significant after correcting for multiple comparisons. While other traits or personality factors, such as caregiving motivation, may play a more influential role in explaining this relationship (Buckels et al. 2015; Hofer et al. 2018), the absence of effects may be due to methodological considerations discussed earlier, rather than a true lack of influence from individual differences. We did, however, find that trait empathic concern was positively associated with caregiver sensitivity, supporting the notion that empathy plays a fundamental role in caregiving behavior (de Waal 2008; De Waal and Preston 2017; Decety et al. 2015).

It is important to note that our findings should be considered in light of the timing of our stress manipulation and measurements of outcome variables. Previous studies have generally examined effects of the TSST on cognitive processes 20–25 min after stressor onset (Plieger and Reuter 2020). In our study, situational empathic behavior was assessed approximately 5–15 min post‐stressor onset. While prior research indicates rapid effects of stress on cognitive processes (de Kloet and Joëls 2023; Hermans et al. 2014), and our perceived stress responses indicate significant increases in subjective stress experiences shortly after the start of our stress manipulation, this timing may affect comparability with previous studies. Similarly, our adaptation of the TSST, which was interrupted by empathy tasks and the interaction with the infant simulator, along with differences in the timing of saliva sample collection, may also limit direct comparisons with earlier studies. The standard TSST typically elicits peak salivary cortisol concentrations between 10 and 20 min after the end of the stress manipulation, which itself lasts 10 min (Kirschbaum et al. 1993). In our study, the stress manipulation was extended by interruptions with the additional tasks, and cortisol concentrations were measured only after the final interaction with the infant simulator. This likely resulted in missing the peak of the cortisol response and instead captured the subsequent decline in cortisol concentrations. Nonetheless, we observed clear differences between conditions, with a stronger decline in cortisol concentrations in the control group, confirming that our stress manipulation was successful. However, this may have reduced our ability to detect effects of individual differences in stress responses.

The generalizability of our findings also warrants careful consideration. First, only female participants were included in the study. Prior research has shown sex differences in response to experimentally induced acute stress, with men generally exhibiting stronger physiological stress responses than women (Plieger and Reuter 2020). Moreover, studies suggest that acute stress may affect empathy differently across sexes. For instance, men with a high cortisol response after the TSST tend to recognize emotions better than women with a similar response (Smeets et al. 2009). Also, acute stress has been found to impair empathetic abilities in women, while having little effect on men (Crenshaw et al. 2019). Our sample additionally consisted of individuals without children and no history of pregnancy, raising further questions about how findings may translate to parents. Both pregnancy and child care may extensively impact caregiver's sensitivity to infants’ signals and needs, likely due to structural brain changes, neural tuning, and hormonal changes (Abraham et al. 2014; Dudek et al. 2020; Hoekzema et al. 2017; Parsons et al. 2017). These considerations highlight the need for future research to include more diverse samples, both in terms of sex and caregiving experience, to better understand potential differences when examining stress effects on empathic behavior and caregiving practices.

## 5 Conclusion

In the current study, we found that acute stress did not affect caregiver sensitivity in a sample of nulliparous women interacting with an infant simulator. Similarly, situational empathic behavior did not appear to mediate the effects of acute stress on caregiver sensitivity. Nonetheless, our findings offer valuable insights into the complex relationship between stress, empathy, and caregiving, highlighting both the possible subtlety of acute stress effects and the methodological challenges involved in detecting them. This emphasizes the need for sensitive instruments capable of detecting such nuanced effects. Moreover, acute stress may produce more subtle outcomes compared to chronic stress or the accumulation of stressors. Continued research that distinguishes between these types of stress will be essential for deepening our understanding of how stress impacts caregiving practices and, consequently, child outcomes.

## Conflicts of Interest

The authors declare no conflicts of interest.

## Supporting information




**Supplementary Information**: dev70177‐sup‐0001‐SuppMat.decx

## Data Availability

The data that support the findings of this study are available from the corresponding author upon reasonable request.
