# Supplementary material for: Navigating Stress: Exploring the Role of Empathy in Caregiving
Source: Dev Psychobiol. 2026 Jun 16;68(4):e70177. doi: 10.1002/dev.70177 (PMC13273094; doi:10.1002/dev.70177)
Supplement: Supplementary file 1 — Supplementary Information: dev70177‐sup‐0001‐SuppMat.decx [file DEV-68-e70177-s001.docx]

**Navigating Stress: Exploring the Role of Empathy in Caregiving Under Stress**

**Supplemental Material**

**Principal Component Analyses**

We performed a Principal Component Analysis (PCA) to explore underlying factors in our measures of empathic behaviour. In order to perform the PCA analyses, we imputed missing data through the estimation of one component using an iterative PCA algorithm with the missMDA package (Josse & Husson, 2016). Data imputation did not affect the distribution of individual measures of empathic behaviour, nor did it affect correlations between these individual measures (see Figures S1 and S2).

| **Figure S1**  *Overview of Distributions of and Associations Between Individual Measures of Empathic Behaviour Before Imputation of Missing Data* |
| --- |
| 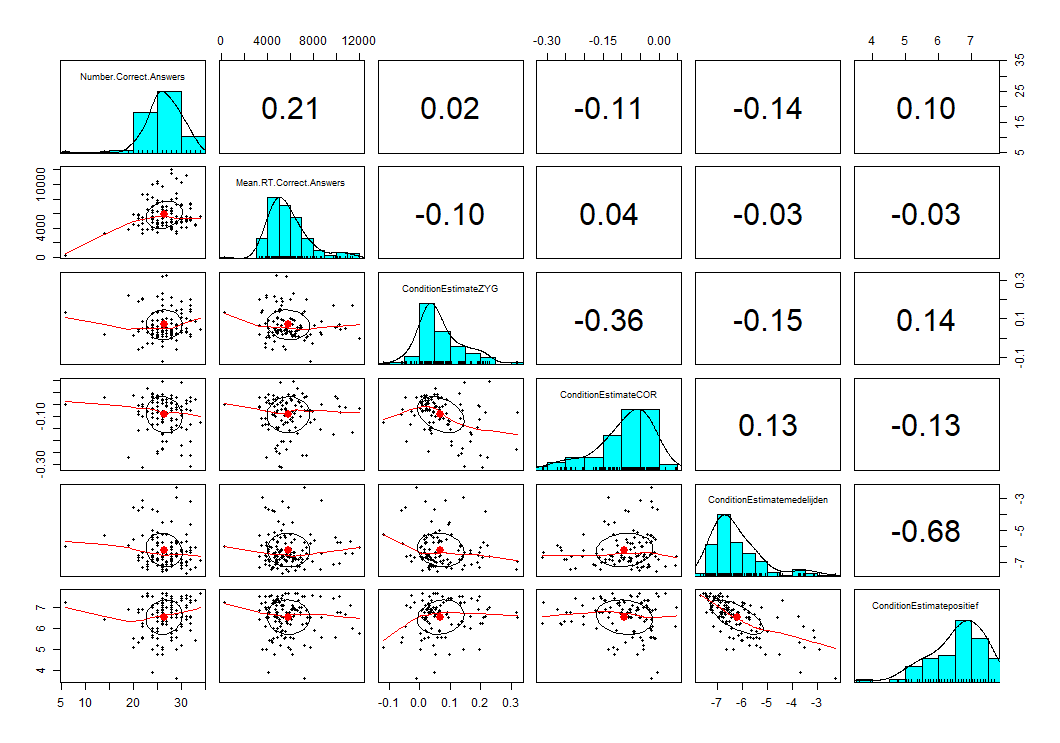 |

| \| **Figure S2**  *Overview of Distributions of and Associations Between Individual Measures of Empathic Behaviour After Imputation of Missing Data* \| \| --- \| \| 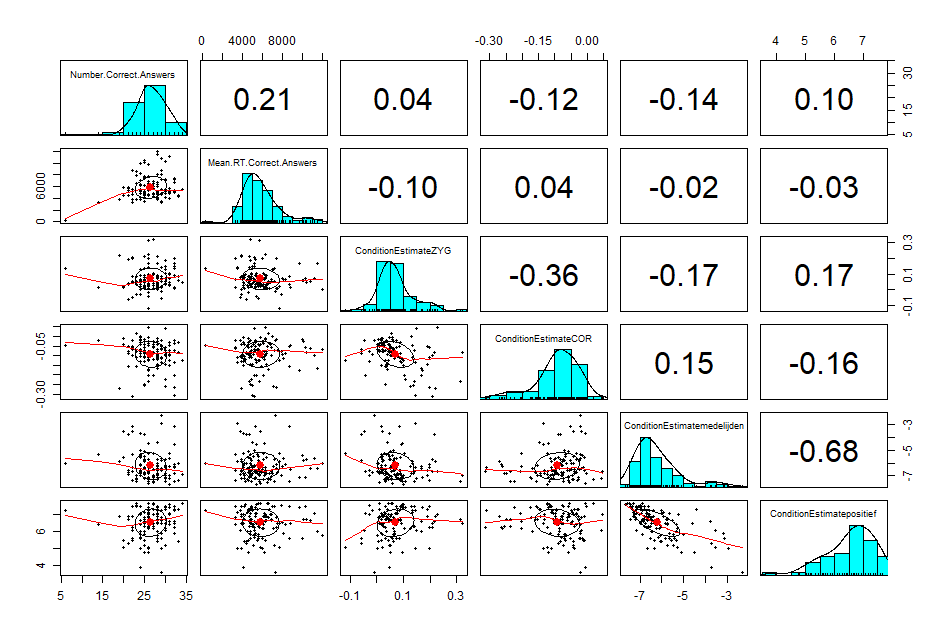 \| |
| --- | --- | --- |

PCA analyses revealed that a total of four components would account for 84.21% of variance in the data (see Figure S3). Although three components had eigenvalues greater than 1, the elbow point in the scree plot is more pronounced after the inclusion of four components than after three (see Figure S4).

| **Figure S3**  Scree Plot of Variance Explained by the Number of Components |
| --- |
| 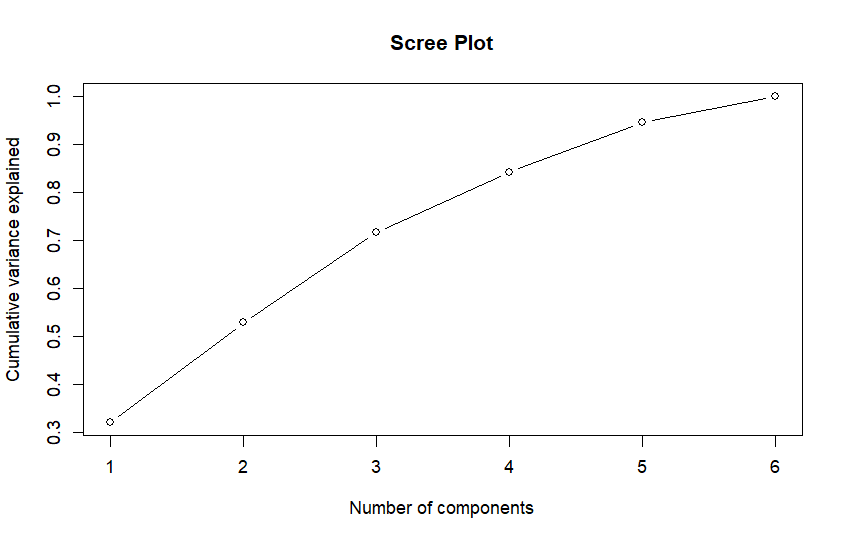 |
| **Figure S4**  *Scree Plot of Eigenvalues per Component.* |
| 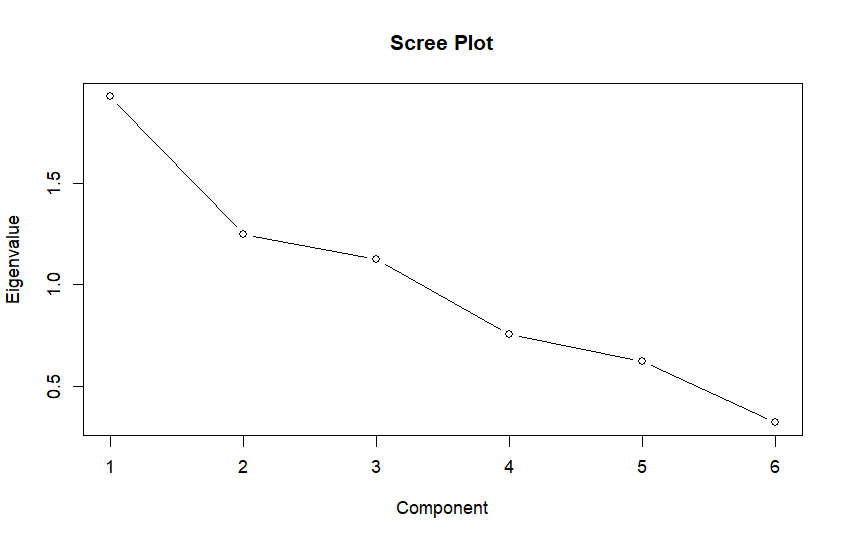 |

Using the psych package (Revelle, 2025), the Kaiser-Meyer- Olkin test estimated that the factor adequacy for most items ranged from miserable to mediocre, with an unacceptable value for mean RT of correct RMET items (see Table S1). The Bartlett’s test ruled out that the variables in the dataset were uncorrelated, *X*^2^ (15) = 112.23, *p* < .001. An additional parallel analyses comparing the factor analyses of the actual data with simulated data, suggested a total of three factors (see Figure S5).

**Table S1**

*Overview of Measure of Sampling Adequacy (MSA) Values for Each Item*

| Item | MSA score (overall MSA = .56) |
| --- | --- |
| Number of correct items RMET | .59 |
| Mean RT for correct items RMET | .49 |
| Condition estimate ZYG activity | .61 |
| Condition estimate COR activity | .60 |
| Condition estimate empathic concern rating | .54 |
| Consition estimate positive affect rating | .54 |

| **Figure S5**  *Comparison of Factor Analyses of Actual Data Against a Parallel Analysis With Simulated Data* |
| --- |
| 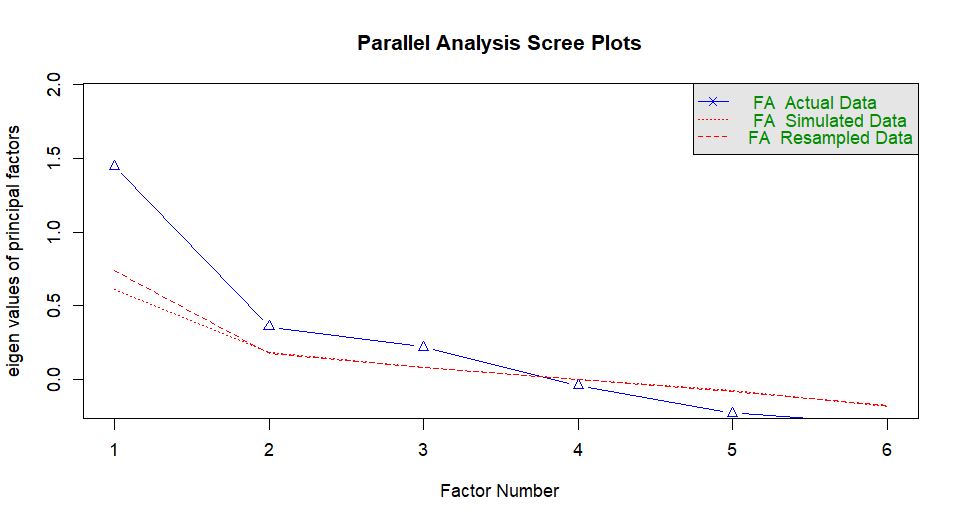 |

Due to the absence of significant inter-factor correlations (see Table S2), we extracted three factors following oblique rotation, which revealed that condition estimates for empathic concern and positive affect ratings loaded most strongly on the first factor, the condition estimates for ZYG and COR muscle activity loaded most strongly on the second factor, and the number of correct responses on the RMET and the mean RT for these correct answers loaded most strongly on the third factor (see Table S3).

| **Table S2**  *Correlations Between Factors* | | | |
| --- | --- | --- | --- |
|  | Factor 1 | Factor 2 | Factor 3 |
| Factor 1 | 1.00 |  |  |
| Factor 2 | -.33 | 1.00 |  |
| Factor 3 | -.03 | -.08 | 1.00 |

| **Table S3**  *Loadings of Items on Factors* | | | | |
| --- | --- | --- | --- | --- |
|  | Factor 1 | Factor 2 | Factor 3 |  |
| Condition estimate empathic concern rating | -.90 |  |  |  |
| Condition estimate positive affect rating | .77 |  |  |  |
| Condition estimate ZYG activity |  | -.41 |  |  |
| Condition estimate COR activity |  | .85 |  |  |
| Number of correct items RMET |  |  | .67 |  |
| Mean RT for correct items RMET |  |  | .32 |  |

Based on this inspection, we determined that a four-factor solution was most appropriate for interpretability within the context of the study. More negative condition estimates for empathic concern ratings and COR activity are associated with enhanced empathic processes, while more positive condition estimates for positive affect ratings and ZYG activity also reflect increased empathy. Accordingly, it was expected that these variables would load positively or negatively on Factor 1 and Factor 2, respectively. For the RMET, enhanced empathic processing was indicated by both an increase in correct responses and a decrease in reaction times (RTs) to those responses. Therefore, we expected these two measures to load in opposite directions on a single factor. However, both loaded positively on the same factor, and furthermore they were positively correlated (i.e., higher accuracy was associated with longer RTs), complicating interpretation if combined. As a result, we proceeded with a four-factor model: (1) empathic ratings, (2) empathic facial reactivity, (3) number of correct items RMET, and (4) mean RT for correct items RMET. To construct the empathic ratings and empathic facial reactivity factors, variable values were first recoded into positive values so that higher values consistently reflected greater empathic abilities, after which the relevant condition estimates were averaged. This resulted in the composite scores used in our main analyses. Correlations between our individual measures of empathic behaviour and composite scores are presented in Table S4. Firstly, these correlations show that our composite scores of empathic ratings and empathic facial reactivity during the affective images task were strongly linked to the empathic processes underlying these scores. ZYG and COR activity were strongly associated with our composite empathic facial reactivity score. Additionally, they were positively correlated, indicating that participants who showed greater differentiation between positive and negative images did so with both muscles (*p*s < .001). Similarly, compassion and positive affect ratings were strongly associated with our composite score of empathic ratings. Also, they were positively correlated, indicating that participants who distinguished more strongly between positive and negative images did so with both subjective ratings (*p*s < .001). During the RMET, the number of correct answers was positively associated with the average response time to correct answers. This indicates that participants who took longer to respond correctly also answered more questions correctly (*p* < .05).

| **Table S4**  *Overview of Pearson Correlations Between Measures* | | | | | | | |
| --- | --- | --- | --- | --- | --- | --- | --- |
| **Measures (scaled)** | **1** | **2** | **3** | **4** | **5** | **6** | **7** |
| 1. Composite score “Empathic facial reactivity” |  |  |  |  |  |  |  |
| 2. ZYG condition estimate | .82*** |  |  |  |  |  |  |
| 3. COR condition estimate | .82*** | .36*** |  |  |  |  |  |
| 4. Composite score “Empathic ratings” | .19 | .16 | .14 |  |  |  |  |
| 5. Compassion ratings | .17 | .15 | .13 | .94*** |  |  |  |
| 6. Positive affect ratings | .17 | .14 | .13 | .89*** | .68*** |  |  |
| 7. Correct items RMET | .07 | .02 | .11 | .13 | .14 | .1 |  |
| 8. RT correct items RMET | -.07 | -.1 | -.04 | 0 | .03 | -.03 | .21* |
| *Note*. * *p* < .05; ** *p* < .01; *** *p* < .001; COR = corrugator supercilia; RMET = Reading the Mind in the Eyes Task; RT = reaction time; ZYG = zygomaticus major | | | | | | | |

**Reference**

Josse, J., & Husson, F. (2016). missMDA: A Package for Handling Missing Values in Multivariate Data Analysis. *Journal of Statistical Software*, *70*(1), 1–31. https://doi.org/10.18637/jss.v070.i01

Revelle, W. (2025). *psych: Procedures for Psychological, Psychometric, and Personality Research*. Northwestern University. https://CRAN.R-project.org/package=psych
